# Computed Tomography slice interpolation in the longitudinal direction based on deep learning techniques: To reduce slice thickness or slice increment without dose increase

**DOI:** 10.1371/journal.pone.0279005

**Published:** 2022-12-15

**Authors:** Shuqiong Wu, Megumi Nakao, Keiho Imanishi, Mitsuhiro Nakamura, Takashi Mizowaki, Tetsuya Matsuda

**Affiliations:** 1 The Institute of Scientific and Industrial Research, Osaka University, Ibaraki, Osaka, Japan; 2 Graduate School of Informatics, Kyoto University, Kyoto, Japan; 3 e-Growth Co., Ltd., Kyoto, Japan; 4 Division of Medical Physics, Department of Information Technology and Medical Engineering, Human Health Sciences, Graduate School of Medicine, Kyoto University, Kyoto, Japan; 5 Department of Radiation Oncology and Image-applied Therapy, Graduate School of Medicine, Kyoto University, Kyoto, Japan; Fudan University - Handan Campus: Fudan University, CHINA

## Abstract

Large slice thickness or slice increment causes information insufficiency of Computed Tomography (CT) data in the longitudinal direction, which degrades the quality of CT-based diagnosis. Traditional approaches such as high-resolution computed tomography (HRCT) and linear interpolation can solve this problem. However, HRCT suffers from dose increase, and linear interpolation causes artifacts. In this study, we propose a deep-learning-based approach to reconstruct densely sliced CT from sparsely sliced CT data without any dose increase. The proposed method reconstructs CT images from neighboring slices using a U-net architecture. To prevent multiple reconstructed slices from influencing one another, we propose a parallel architecture in which multiple U-net architectures work independently. Moreover, for a specific organ (i.e., the liver), we propose a range-clip technique to improve reconstruction quality, which enhances the learning of CT values within this organ by enlarging the range of the training data. CT data from 130 patients were collected, with 80% used for training and the remaining 20% used for testing. Experiments showed that our parallel U-net architecture reduced the mean absolute error of CT values in the reconstructed slices by 22.05%, and also reduced the incidence of artifacts around the boundaries of target organs, compared with linear interpolation. Further improvements of 15.12%, 11.04%, 10.94%, and 10.63% were achieved for the liver, left kidney, right kidney, and stomach, respectively, using the proposed range-clip algorithm. Also, we compared the proposed architecture with original U-net method, and the experimental results demonstrated the superiority of our approach.

## 1 Introduction

In recent years, machine learning algorithms such as convolutional neural networks (CNNs), the U-net architecture, and generative adversarial networks have been widely applied to image processing and pattern recognition [1–4]. As they have become more popular, researchers have started to apply them to Computed Tomography (CT) image processing [5–10]. For example, Gupta et al. proposed a CNN-based algorithm that uses neural networks to replace the projector in sparse-view CT reconstruction [6]. Similarly, U-net architecture has recently been applied to sparse-view reconstruction of CT images [8]. Besides sparse-view reconstruction, super-resolution reconstruction of CT data is also a popular research topic [14].

As we know, many famous methods for super-resolution imaging have been proposed recently [11–14]. Some of them especially deep-learning-based ones have been already applied to CT data, which improved the image resolution (from less to more pixels) of 2D CT slice in the axial plane [14]. When we compared these super-resolution imaging techniques with the main issue we mentioned above (sparsely sliced CT problem), we found that they have one thing in common. The super-resolution imaging aims at improving the resolution in the axial plane by creating more pixels, while our goal is to improve the resolution in the longitudinal direction by creating more slices (densely sliced CT reconstruction from sparsely sliced CT data). Although creating a whole slice is much more difficult than creating a new pixel, this kind of study would be worthwhile because longitudinal resolution plays an important role on disease diagnosis [15, 16]. However, we found that there are rare researches related to deep-learning-based resolution enhancement in the longitudinal direction. Park et al. used a 3D deep network to reduce the slice thickness. However, their model has fixed patterns and is difficult to be applied to other data or applications [17]. In our previous research, we used the U-net architecture to reconstruct one middle CT slice from two neighboring slices [18]. Our results showed that the U-net architecture outperformed linear interpolation in terms of mean absolute error (MAE). However, the simple network architecture used in the previous work allowed only one middle slice to be reconstructed [18]. This limits its use in real applications, in which two or more slices may be required to be reconstructed.

In this study, we propose a deep-learning-based framework which can achieve densely sliced CT reconstruction in the longitudinal direction without any dose increase. Differently from our previous work [18], the proposed approach adopts a parallel architecture that could reconstruct multiple new slices. It contains multiple networks with U-net architecture [2]. These U-net architectures share the same input but output different CT slices. As a variant of CNNs, the U-net architecture has been widely used in medical image segmentation [19–24]. Its layers are arranged into a U-shaped architecture, containing down-sampling and up-sampling processes. It extracts both global and local features from the input image [2], improving performance in processing medical images [25]. The parallel architecture is proposed to create multiple CT slices and prevent them from influencing one another.

Moreover, we have found that most approaches process one CT slice as a whole unit during image reconstruction [6]. However, for a specified organ, learning only the ROI (region of interest) of the organ may be more effective than learning the whole CT slice. Therefore, we also propose a range-clip technique that improves the reconstruction quality of a specified organ by enlarging the range of CT values inside this organ during the training process. The range is computed from training data alone, so it is not necessary to label the test data. This technique aims to enhance learning in the ROI of the specified organ.

**Fig 1:**
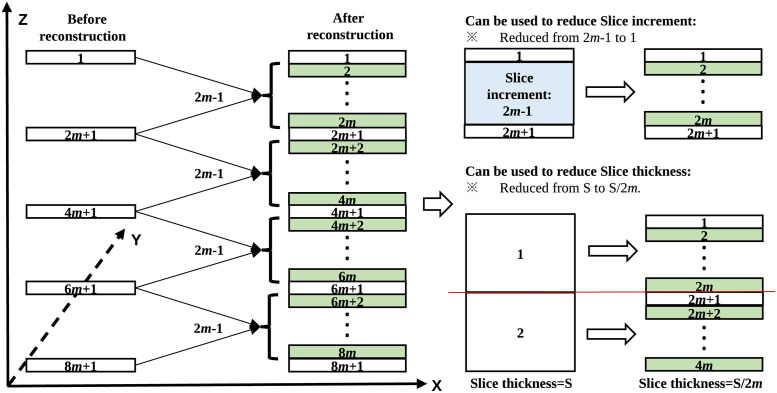
CT slice reconstruction based on the proposed approach that reconstructs 2*m* − 1 middle slices from two adjacent slices. The green slices are newly reconstructed ones. Then we copy the original slice as the first slice of the reconstructed data to generate the whole CT data.

Note that the proposed parallel architecture for densely sliced CT reconstruction and range-clip technique for organ enhancement are two independent methods. However, experiment results showed that a combination of the two proposed algorithms achieved the best performance. This combination achieved densely sliced CT reconstruction in the longitudinal direction without changing any CT device parameters such as slice thickness or pitch. Fig 1 shows the proposed CT slice reconstruction process, which has two contributions: (1) to reconstruct densely sliced CT to improve the resolution in the longitudinal direction without any dose increase, and (2) to interpolate middle slices to fill the gap between two adjacent CT slices in the case that slice increment is larger than slice thickness.

## 2 Materials and methods

In this section, we first describe the proposed parallel U-net architecture for CT slice reconstruction in the longitudinal direction, and then explains the range-clip technique for improving the reconstruction quality of each specified organ.

### 2.1 Parallel U-net architecture

**Fig 2:**
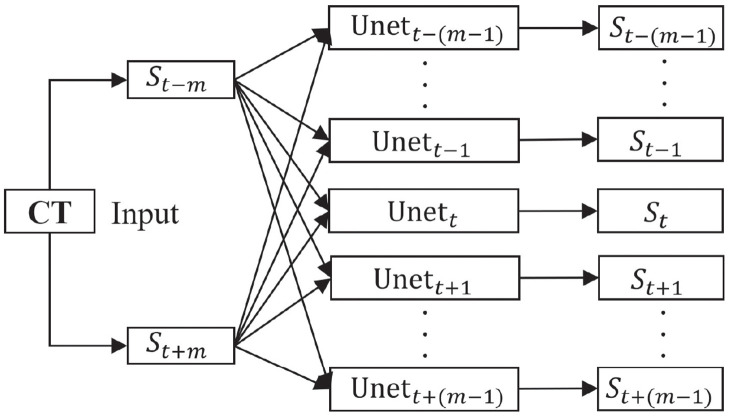
Architecture of the proposed approach for CT slice reconstruction. The neural networks deduces multiple middle slices from two neighboring slices. A parallel architecture is adopted to prevent the different target slices from influencing one another, so that the target slices are computed separately.

Suppose we reconstruct 2*m* − 1 middle slices from pairs of adjacent slices. Let sequence *φ* represent the target dense CT slices after reconstruction, i.e., *φ* = {*S*_1_, *S*_2_, …, *S*_*t−m*_, *S*_*t*−(*m*−1)_, …, *S*_*t*_, …, *S*_*t+m*−1_, *S*_*t+m*_, …, *S*_*n*_} where *t* is the index of the sorted slices and *n* is the total number of dense CT slices. The proposed approach is to reconstruct {*S*_*t*−(*m*−1)_, …, *S*_*t*_, …, *S*_*t*+*m*−1_} from *S*_*t*−*m*_ and *S*_*t*+*m*_ to achieve the densely sliced CT reconstruction in the longitudinal direction. The architecture of the proposed reconstruction algorithm is shown in Fig 2.

**Fig 3:**
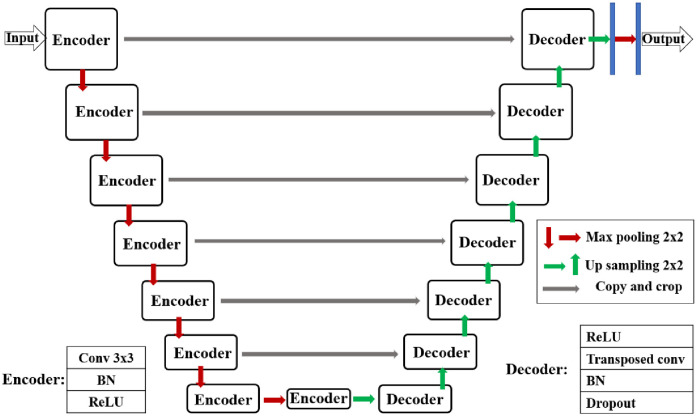
Outline of layers of each U-net shown in Fig 2. BN here means batch normalization layer.

To prevent the different reconstructed slices from influencing one another, we adopt a parallel architecture with multiple U-net architectures. These U-net architectures are each used to reconstruct its own target CT slice, using the same input slices. The image size of both the input and output slices is 400 × 320 pixels. Each U-net architecture contains eight encoder and seven decoder modules, respectively, as shown in Fig 3. A dropout layer is added in the last layer of decoder module to reduce the influence of over-fitting.

We also implemented a learning technique that uses one U-net architecture to reconstruct multiple slices. However, its accuracy was lower than that of the proposed parallel architecture (comparison shown in the Results section). Compared with the original U-net method, the proposed parallel approach learns the nonlinear relationships among multiple neighborhood slices using multiple sets of parameters. As we mentioned above, the proposed architecture can be used either to interpolate the middle slides to fill the gap, or to reconstruct slices to reduce the slice thickness.

### 2.2 Organ-oriented reconstruction

In this section, we discuss slice reconstruction for a specific organ, which we name organ-oriented reconstruction. The range of CT values across all human organs is wide. However, for a certain organ, e.g. the liver, the range can be much narrower. It is possible to improve the reconstruction quality of a certain organ if we focus on the range within it and then enlarge this range. In this study, we used a CT dataset in which the ROI of each organ had been labeled by board-certified radiation oncologists. Based on these labeled data, we obtained an approximate distribution of the CT values for each organ and then assessed the organ’s range.

**Fig 4:**
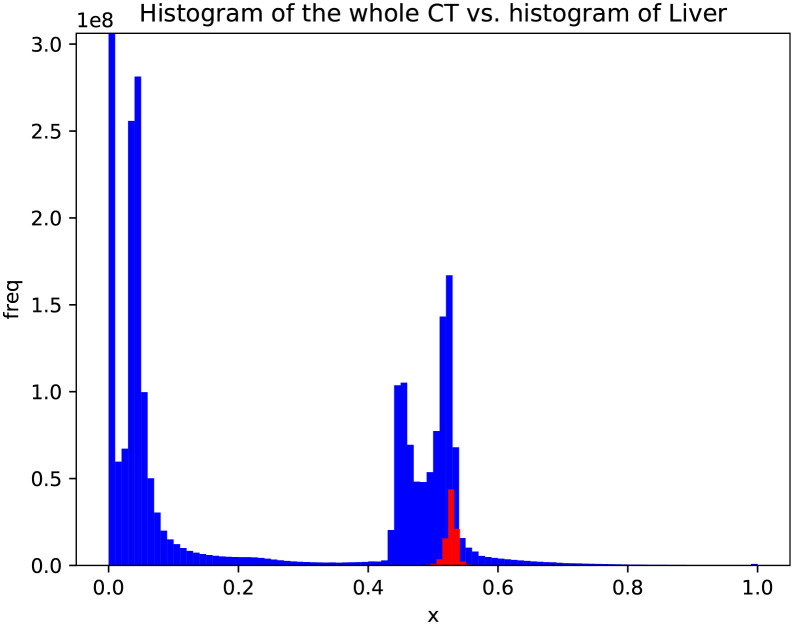
Histograms of the whole CT image and the liver alone. The blue and red areas show the distributions of the CT values across the whole slice and inside the liver. The X-axis is the normalized CT values, and the Y-axis is the frequency.

Fig 4 shows the CT value range of the liver compared with that of the whole slice in the training dataset. There may be foreign objects such as drainage tubes or surgical staples inside the body, introducing noise to the distribution. Therefore, we manually checked the distribution for each organ. If noise was present, we removed it with filters manually. The range of all CT values across the training data was normalized to [0, 1], and then the range [*R*_*ll*_, *R*_*lh*_] of CT values of pixels inside the liver was computed based on the labeled training data (0 ≤ *R*_*ll*_ ≤ 1, 0 ≤ *R*_*lh*_ ≤ 1, and *R*_*ll*_ ≤ *R*_*lh*_). Finally, a linear function *f*(*x*) was established to change the CT value for each pixel x, enlarging the range of the liver from [*R*_*ll*_, *R*_*lh*_] to [0, 1]. The transformation function *f*(*x*) is defined as

$$f\left(x\right)=\{\begin{array}{cc} 0 & \text{if}\;x<R_{ll}\\ 1 & \text{if}\;x>R_{lh}\\ \text{B}\left(x-\text{C}\right) & \text{if}\;R_{ll}\leq x\leq R_{lh} \end{array}$$ (1)

In (1), C and B are constants that ensure that *f*(*x*) falls within the range of [0, 1] for liver pixels. C and B are exclusively based on the training data, and their values change when the range-clip is applied to other organs. Because the range of the liver is enlarged, the quality of this region was improved after the range-clip processing. Although other regions outside the liver may lose information when the above range-clip processing is performed, this did not influence the final results because we also trained a model based on the original data to save the information about non-liver regions. In other words, we trained the networks for reconstructing the liver and non-liver regions using the range-clipped and original data, respectively. Finally, we merged the outputs of both trained networks. To maintain consistency during merging, we applied *f* to the test data before inputting them into the trained networks and *f*^−1^ to the network’s output.

**Fig 5:**
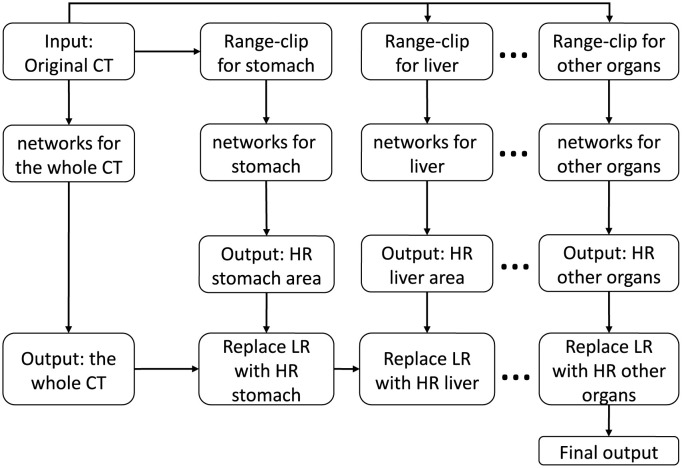
Training process of organ-oriented CT reconstruction. Each neural networks is trained for each specified organ. The organ areas in the original output are then replaced with the results of these organ-oriented networks. HR: high-quality result; LR: low-quality result. Here networks means the proposed parallel U-net architecture shown in Fig 2.

Similar to that for the liver, we can implement the proposed range-clip-based learning method for other organs (e.g., lungs, kidneys, stomach). The designed training process is shown in Fig 5. The range-clip technique aims to enhance learning within a specified organ by exploiting the range features of the training data. It provides a chance to improve the reconstruction quality by labeling the training data rather than the test data. The air contained in the stomach occasionally influences the reconstruction quality. Therefore, we used filters to exclude air areas inside the stomach before the organ-oriented CT reconstruction was conducted.

In summary, The organ-oriented CT reconstruction is a combination of parallel U-net architecture and range-clip technique, improving the learning of pixels within a specific organ by exploiting its range feature.

### 2.3 Database detail

Our database contains 130 CT data from 130 patients. The CT data had a resolution of 1.0742 × 1.0742 × 2.5*mm*^3^, and their range was clipped to [−1000, 1000] HU (Hounsfield units) for observing soft organs. Areas outside this range such as bones were not considered in this study. For each CT series, labels indicating the liver, left kidney, right kidney, and stomach regions were provided by board-certified radiation oncologists.

The proposed study is retrospective, and the patient data were obtained from Kyoto University Hospital, Kyoto, Japan. There was no specific inclusion/exclusion criteria for the participants. Patients participated voluntarily. Before providing their data, a written consent form which included the introduction of the research, the description of the data detail, and the agreement statement was obtained from each participant. In addition, we allowed opt out for all participants.

This study followed all dictates of the Declaration of Helsinki and the Ethics Review Board of Kyoto University Hospital, and the Faculty of Medicine approved the research (approval number R1446).

## 3 Results

In this section, first we describe the experimental settings, and then introduce the evaluation of the proposed parallel U-net architecture, and finally show the evaluation of the proposed organ-oriented CT reconstruction.

### 3.1 Experimental settings

In this subsection, we describe the criteria used for performance evaluation, the experimental parameters, and the properties of the data. In radiotherapy, errors in both CT values and organ layouts influence the accuracy of dose calculation. Thus, we evaluated the proposed approaches based on both the observed CT values and organ appearance. We used MAE to evaluate the performance of CT value reconstruction and SSIM (structural similarity [26, 27]) to evaluate the appearance of the reconstructed slices.

In experiments, we evaluated the models’ performance at reconstructing one, two, three, four, and five middle slices from each pair of adjacent slices (*m* = {1, 1.5, 2, 2.5, 3}, so that 2*m* − 1 = {1, 2, 3, 4, 5}). How to decide the *m* value will be explained in the Discussion section. The values of C and B in (1) for different organs are shown in Table 3, which were decided by labeled training data.

Using the proposed method, we can reconstruct multiple middle slices from any pair of adjacent slices. However, after reconstruction, the ground truth data need to correspond with the input data if we intend to evaluate the proposed algorithm. To generate the ground truth data, we used the CT data with all slices as the densely sliced CT series and created sparse versions for performance evaluation. Suppose that *m* = 3, which means that five slices are reconstructed from two adjacent slices. We separate each original CT into two groups: *φ*_1_ = {*S*_1_, *S*_7_, *S*_13_, …, *S*_*n*−12_, *S*_*n*−6_, *S*_*n*_} and *φ*_2_ = {*S*_2_, *S*_3_, *S*_4_, *S*_5_, *S*_6_, …, *S*_8_, *S*_9_, *S*_10_, *S*_11_, *S*_12_, …, *S*_*n*−5_, *S*_*n*−4_, *S*_*n*−3_, *S*_*n*−2_, *S*_*n*−1_}, where *φ*_1_ is the sparse version and *φ*_2_ is the reconstructed target slices. Finally, the densely sliced CT series can be reconstructed by merging *φ*_1_ and *φ*_2_. For other values of *m*, we can regroup the data for the same evaluation.

For training, *φ*_1_ and *φ*_2_ were used as the input and output of the proposed parallel U-net architecture, respectively. For testing, *φ*_1_ was used as the input to the neural network, and *φ*_2_ was used as the ground truth for evaluation. By comparing *φ*_2_ with the output of the trained network in test mode, we can evaluate the proposed approaches’ reconstruction performance. For more precise evaluation (e.g., to evaluate the reconstruction of nonexistent CT data with slice thickness of less than 0.5 mm), clinical measurements by professional doctors are required (see details in the Discussion section). Note that we used 80% (104 cases) of the data for training, and the remaining 20% (26 cases) for testing in this study.

In this research, we used multiple U-nets architecture, which requires more GPU resources than the single U-net one in the training phase. In our experiments, we used GeForce RTX 2080 whose memory is 11GB for training each U-net. We trained the multiple U-nets parallel using multiple GPUs simultaneously, and it took about one hour. However, in the test, we only used one GPU.

### 3.2 Evaluation of the proposed parallel U-net architecture-based CT slice reconstruction

First, we evaluated the performance of the proposed parallel U-net architecture at reconstructing one, two, three, four, and five (*m* = {1, 1.5, 2, 2.5, 3}) middle slices from pairs of adjacent slices. Table 1 summaries the results: we found that *m* = 1 had the lowest MAE, whereas *m* = 3 had the highest MAE. In the following experiments, we use *m* = 3 as an example to compare the proposed approach with other methods because *m* = 3 is the most challenging setting. The MAE with *m* = 2 was slightly smaller than that with *m* = 1.5. We believe that the training of *m* = 1.5 may have been influenced by the bias of the training data. However, MAE tended to increase as the *m* value increased.

**Table 1:** Mean absolute error (MAE) of the reconstruction of one, two, three, four, and five slices by the proposed parallel U-net architecture.

The *m* value	*m* = 1	*m* = 1.5	*m* = 2	*m* = 2.5	*m* = 3
Number of target slices (2*m* − 1)	2*m* − 1 = 1	2*m* − 1 = 2	2*m* − 1 = 3	2*m* − 1 = 4	2*m* − 1 = 5
MAE (mean absolute error: HU)	10.55	15.89	15.07	16.43	18.03

**Fig 6:**
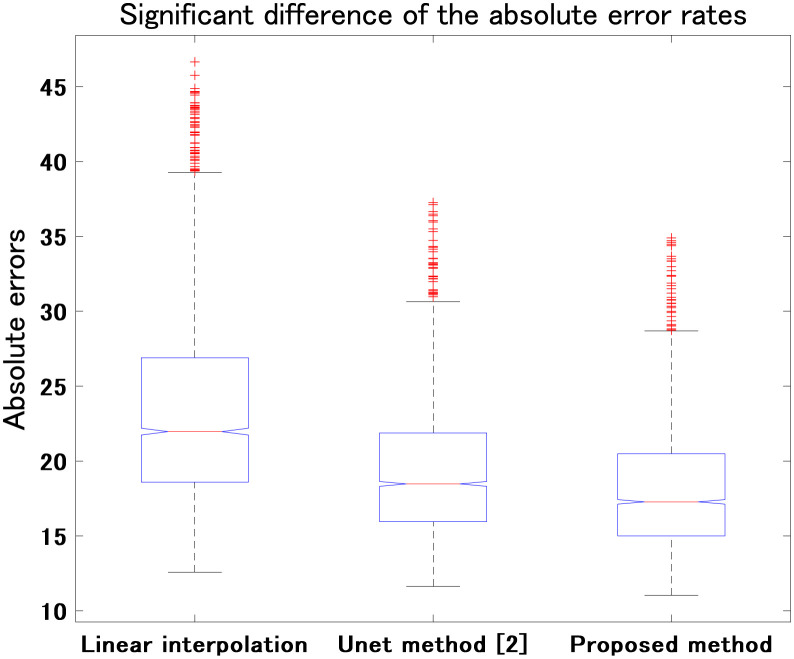
Comparison among linear interpolation, the other U-net method [2], and the proposed parallel U-net architecture (all *p* < 0.01). Vertical axis shows the absolute errors of the reconstructed slices.

Second, we compared the proposed parallel U-net architecture with conventional linear interpolation and the other method using one U-net architecture [2] to produce multiple slices when *m* = 3. Table 2 presents a comparison between all three methods’ results for all reconstructed CT slices. The proposed approach reduces the MAE by 22.05% and 6.09% compared with linear interpolation and the other U-net method [2], respectively. Fig 6 shows the significant differences between the MAE values given by the proposed approach, linear interpolation, and the other U-net method [2] (all *p* < 0.01). The results demonstrate that a significant improvement is achieved by the proposed parallel U-net architecture compared with both linear interpolation and the other U-net method [2]. Compared with the case that uses one U-net architecture to reconstruct multiple slices, the proposed parallel strategy is better because learning parameters to fit one slice is easier than to fit multiple slices.

**Table 2:** Comparison between linear interpolation, U-net architecture [2], and the proposed parallel U-net architecture: The bold numbers show the best results.

MAE (mean absolute error: HU); *m* = 3	Slice 1	Slice 2	Slice 3	Slice 4	Slice 5	Average
Linear interpolation	18.11	25.75	27.87	25.77	18.16	23.13
U-net architecture [2]	16.18	20.78	21.92	**21.11**	16.03	19.20
Proposed parallel U-net architecture	**15.60**	**18.67**	**20.04**	21.50	**14.34**	**18.03**
Improvement (Proposed vs. U-net)	3.58%	10.15%	8.58%	-1.85%	10.54%	6.11%
Improvement (Proposed vs. Interpolation)	13.86%	27.50%	28.09%	16.57%	21.04%	22.06%

### 3.3 Evaluation of the proposed organ-oriented CT slice reconstruction

**Fig 7:**
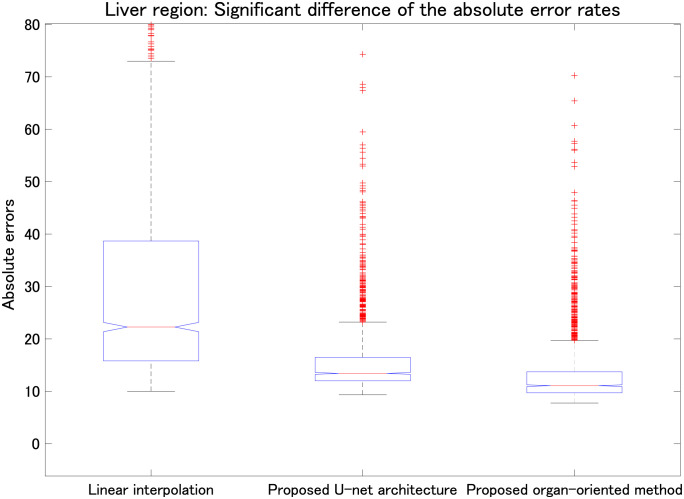
Comparison of linear interpolation, the proposed parallel U-net architecture, and the proposed organ-oriented method (all *p* < 0.01). Vertical axis shows the absolute errors of the liver region in the reconstructed slices.

**Fig 8:**
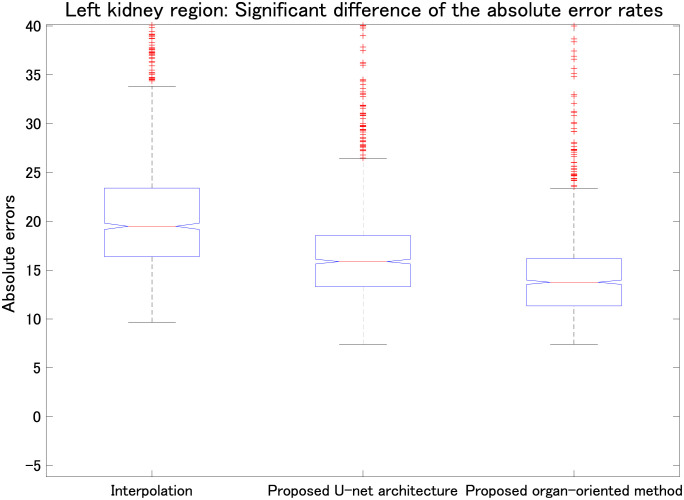
Comparison among linear interpolation, the proposed parallel U-net architecture, and the proposed organ-oriented method (all *p* < 0.01). Vertical axis shows the absolute errors of the left kidney region in the reconstructed slices.

**Fig 9:**
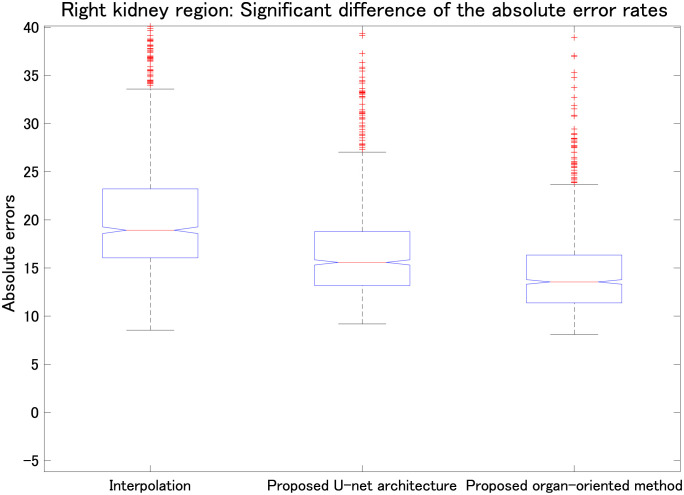
Comparison among linear interpolation, the proposed parallel U-net architecture, and the proposed organ-oriented method (all *p* < 0.01). Vertical axis shows the absolute errors of the right kidney region in the reconstructed slices.

**Fig 10:**
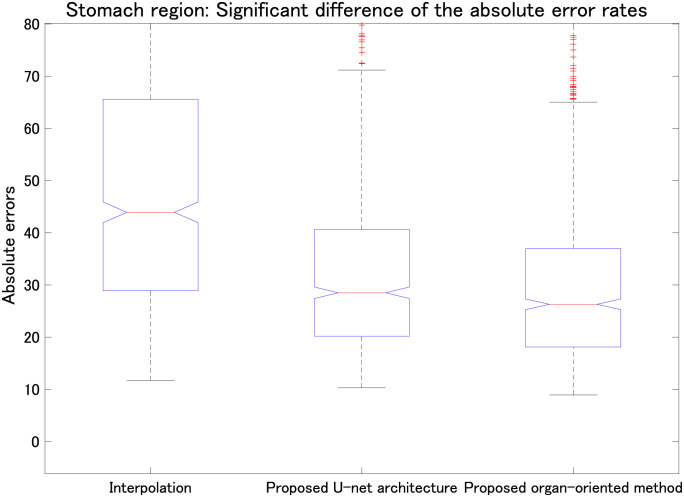
Comparison among linear interpolation, the proposed parallel U-net architecture, and the proposed organ-oriented method (all *p* < 0.01). Vertical axis shows the absolute errors of the stomach region in the reconstructed slices.

We also evaluated the proposed parallel U-net architecture combined with the proposed range-clip technique. Table 3 summarizes the MAE results for the liver, left kidney, right kidney, and stomach, and Table 4 presents the SSIM results for the same organs. To highlight the differences between the computed results and the ground truth, we implemented data normalization before calculating the SSIM values for each slice. Tables 3 and 4 show that the proposed parallel U-net architecture combined with the proposed range-clip technique achieved the best results. Figs 7–10 show the significant differences of linear interpolation, the proposed parallel U-net architecture, and the proposed organ-oriented method for the reconstruction of liver, left kidney, right kidney, and stomach regions, respectively. From these figures, we can conclude that the proposed parallel U-net architecture significantly outperforms the linear interpolation. Then, the proposed organ-oriented method furthermore improves the reconstruction quality for all the specified organs.

**Table 3:** Mean absolute error (MAE): Comparison between the proposed U-net architecture-based method and the organ-oriented approach.

MAE (mean absolute error: HU); *m* = 3	Parameters	Slice 1	Slice 2	Slice 3	Slice 4	Slice 5
Proposed parallel U-net architecture:liver	N/A	14.25	13.24	13.90	14.66	13.50
Proposed organ-oriented method:liver	C = 0.400;B = 4.545	10.91	12.07	12.87	12.14	10.97
Improvement for liver	N/A	23.44%	8.84%	7.41%	17.19%	18.74%
Proposed parallel U-net architecture:left kidney	N/A	15.52	15.86	17.48	16.97	13.27
Proposed organ-oriented method:left kidney	C = 0.418;B = 4.762	12.95	14.53	15.51	14.99	12.30
Improvement for left kidney	N/A	16.56%	8.39%	11.27%	11.67%	7.31%
Proposed parallel U-net architecture:right kidney	N/A	15.63	15.84	17.43	16.61	13.53
Proposed organ-oriented method:right kidney	C = 0.440;B = 6.250	12.80	14.54	15.24	14.98	12.72
Improvement for right kidney	N/A	18.11%	8.21%	12.56%	9.81%	5.99%
Proposed parallel U-net architecture:stomach	N/A	26.14	33.52	37.84	35.15	25.46
Proposed organ-oriented method:stomach	C = 0.320;B = 4.000	23.60	31.48	34.20	26.62	24.58
Improvement for stomach	N/A	9.72%	6.09%	9.62%	24.27%	3.46%

**Table 4:** Structural similarity (SSIM): Comparison between the proposed U-net architecture-based method and the organ-oriented approach.

SSIM (Structral similarity); *m* = 3	Slice 1	Slice 2	Slice 3	Slice 4	Slice 5
Proposed parallel U-net architecture: liver	0.866	0.822	0.812	0.780	0.884
Proposed organ-oriented method: liver	0.885	0.839	0.821	0.810	0.883
Improvement for liver	2.19%	2.07%	1.11%	3.85%	-0.11%
Proposed parallel U-net architecture: left kidney	0.511	0.382	0.353	0.320	0.534
Proposed organ-oriented method: left kidney	0.542	0.411	0.384	0.391	0.552
Improvement for left kidney	6.07%	7.59%	8.78%	22.19%	3.37%
Proposed parallel U-net architecture: right kidney	0.512	0.387	0.360	0.328	0.539
Proposed organ-oriented method: right kidney	0.542	0.421	0.393	0.394	0.552
Improvement for right kidney	5.86%	8.79%	9.17%	20.12%	2.41%
Proposed parallel U-net architecture: stomach	0.616	0.493	0.469	0.440	0.641
Proposed organ-oriented method: stomach	0.637	0.511	0.485	0.490	0.649
Improvement for stomach	3.41%	3.65%	3.41%	11.36%	1.25%

**Fig 11:**
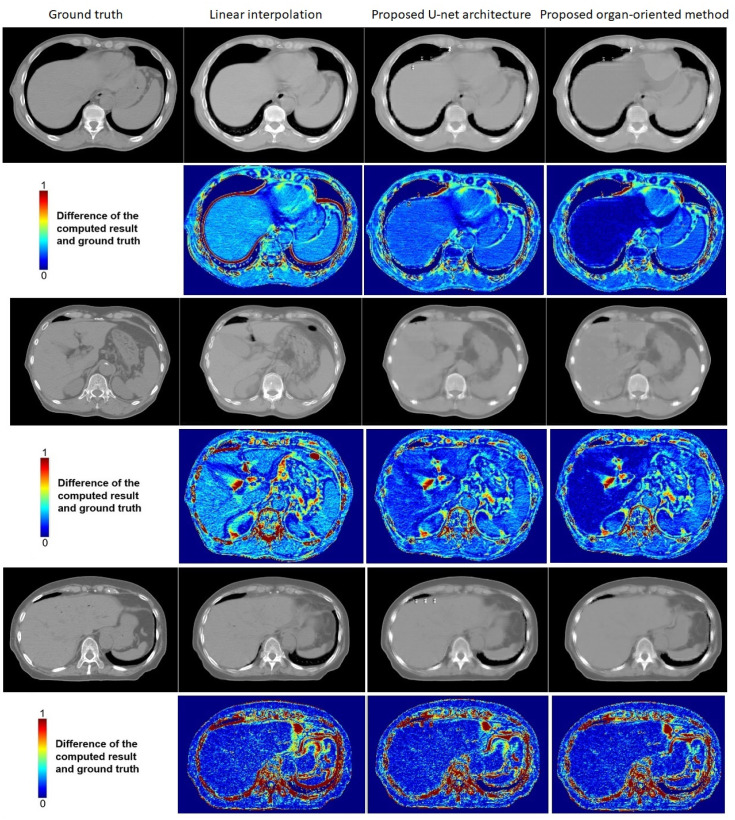
Examples of the comparison among the proposed U-net architecture-based approach, the proposed organ-oriented method, and linear interpolation. From left to right, the images shown in grayscale are the ground truth, the result computed by linear interpolation, the result deduced by the proposed parallel U-net architecture, and the result obtained by the proposed organ-oriented reconstruction. The images shown in color maps are difference images between the compared methods’ results and the ground truth. In the color maps, dark blue indicates the smallest difference and red indicated the largest difference. The window size is adjusted to [−400, 400] for soft tissues.

**Fig 12:**
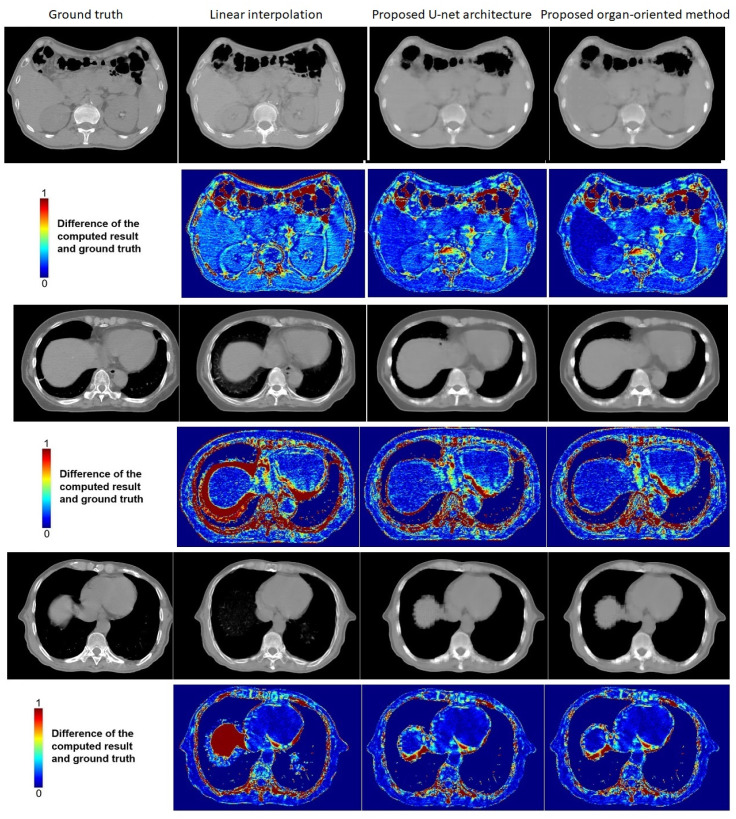
Examples of the comparison among the proposed U-net architecture-based approach, the proposed organ-oriented method, and linear interpolation. From left to right, the images shown in grayscale are the ground truth, the result computed by linear interpolation, the result deduced by the proposed parallel U-net architecture, and the result obtained by the proposed organ-oriented reconstruction. The images shown in color maps are difference images between the compared methods’ results and the ground truth. In the color maps, dark blue indicates the smallest difference and red indicated the largest difference. The window size is adjusted to [−400, 400] for soft tissues.

Figs 11 and 12 shows some examples of comparisons of the proposed organ-oriented reconstruction and parallel U-net architecture-based reconstruction with the reconstruction of linear interpolation. The window size in Figs 11 and 12 was adjusted to [−400, 400] for soft tissues.

A comparison of the slices reconstructed by the proposed methods with the ground truth shows that some details are lost. This is because we reconstructed CT slices that originally did not exist at all, leading to rarity of information to facilitate detail reconstruction. However, the slices reconstructed by the proposed methods are of higher quality than those created by linear interpolation. Although the results of linear interpolation contain many details, they are artifacts, which caused the blur of organ boundaries, and consequently leading to incorrect dose calculation. In contrast, the proposed parallel U-net architecture reduces these artifacts, and the proposed organ-oriented method achieves a further improvement. The combination of the two proposed methods achieves more correct boundaries than linear interpolation, which is important to dose calculation in radiotherapy surgeries. When we compare the difference color-maps, we find that the proposed method also improves the reconstruction quality inside the organs. Although obtaining slice reconstruction results that exactly match the ground truth is impossible, the proposed method reduces the artifacts around each organ more effectively than linear interpolation does. Rows 3–6 in Fig 12 shows that the proposed method can reconstruct the shape of organs in situations where linear interpolation fails.

## 4 Discussion

In this section, we discuss several issues related to the proposed study.

### 4.1 Limitations of the proposed approach

The proposed method cannot be evaluated for reconstructing CT data with slice thickness of less than 0.5 mm because we do not have ground truth data for comparison. However, evaluations based on clinical measurements during surgery are possible. In future work, we plan to invite professional doctors to evaluate the proposed method in clinical situations. Another limitation is that some details inside the organs are lost in the reconstructed CT slices. This is because reconstructing data which do originally not exist is difficult. However, the proposed approach could provide more precise organ boundaries compared with other methods, which can still contribute to dose calculation.

### 4.2 How to decide the value of *m* in Fig 1

The larger the *m* value is, the more middle slices are reconstructed, and the more densely sliced in the longitude direction. Nevertheless, noise also increases as the spatial resolution of CT data is improved [28]. To facilitate a valid reconstruction, we need to make a trade-off between the *m* value and the magnitude of pixel-wise noise. In this study, we simply set a threshold *μ* to restrain the *m* value: when the maximum pixel-wise MAE exceeded *μ*, the increase of *m* was stopped.

### 4.3 Person-dependent vs. Person-independent learning

The proposed approach for CT slice reconstruction is person-independent, which means that the range of each organ used in the proposed range-clip method is an approximation rather than a precise value. This is because we used the pixel values within the organs of one group of persons to predict the range for other different persons. However, based on the proposed approach, we could develop a person-dependent model to achieve more precise ranges for organs by simply changing the types of training data. Both person-independent and person-dependent learning methods have their own advantages. In person-independent learning, it saves time because we can use one model to deal with all samples. On contrary, we can achieve more accurate ranges of each organ in person-dependent learning but need more time in training because we need to change training data for each sample. Since person-dependent reconstruction would entail a different research objective from this study, we will pursue this in our future work.

### 4.4 Supervised-learning vs. self-learning

As we mentioned above, the proposed approach is based on U-net, which is a supervised learning approach [2]. It has an encoding and decoding structure, and requires the labeled data for training. We noticed that both the input and output of the proposed networks are from the same CT data. The purpose of the proposed architecture is to let the networks learn the relationships and regulations in the target itself, which is similar to self-learning. Self-learning is an unsupervised-learning, which is widely used in image clustering [29, 30]. It exploits the similarity among samples and discrepancy between clusters to improve the clustering performance [30]. In recent years, self-learning was also applied to CT data processing [31–33]. For example, Xie et al. proposed a through-plane resolution CT imaging method based on CycleGAN [31]. Fung et al. used self-supervised learning models for COVID-19 lung CT segmentation [32]. Niu et al. applied self-learning to reduce the noise of CT data [33]. These self-learning approaches did not need labeled data for training. However, it maybe more difficult for these methods to guarantee the quality of reconstruction than those supervised learning algorithms whose models can learn directly from the labeled data. Because we have the labeled data for training, we adopted supervised learning in our research. Nevertheless, it will be an interesting research to compare the proposed supervised learning with some self-learning approaches such as CycleGAN [31]. We plan to do this kind of comparisons in our future work.

### 4.5 A comparison with peer work

We compare the proposed approach with previously published works to show a difference. There was a similar research study in which CT was reconstructed from large to small values of slice thickness. It used 3D CNNs to reconstruct CT data from 3-mm (or 5-mm) to 1-mm slice thickness [17]. There were only two patterns of CT reconstruction: 3-mm to 1-mm, and 5-mm to 1-mm. Unlike that work, the proposed approach improves the longitudinal direction resolution by reconstructing internal slices between pairs of adjacent slices. Our approach is based on 2D reconstruction of multiple slices. The number of target slices to be reconstructed is not fixed, which makes the method more suitable for real applications. Furthermore, the proposed method can be used for either large-to-small slice-thickness reconstruction or large-to-small slice-increment reconstruction as shown in Fig 1.

### 4.6 Overlap issue

In the subsection, we discuss an interesting issue that two original slices overlap. In that case, we cannot reduce the slice increment directly. However, we can reduce the slice thickness first, and then the original overlapped slices can become non-overlap ones. Then we can reduce the slice thickness further or increment to improve the resolution in the longitudinal direction using the proposed approach.

## 5 Conclusion

In summary, we proposed a parallel U-net architecture to reconstruct CT slices from neighboring slices. Experimental results demonstrate that this reconstruction is valid, with the U-net architecture playing an important role. Moreover, we proposed a range-clip technique to refine the reconstruction for each specified organ. The proposed approach could be used to reduce the slice thickness or slice increment without any dose increase.

## Supporting information

S1 Dataset (RAR)
